# Post transplant renal vein thrombosis, with successful thrombectomy and review of the literature

**DOI:** 10.1016/j.ijscr.2019.07.066

**Published:** 2019-07-31

**Authors:** Mark Lerman, Matthew Mulloy, Christie Gooden, Salman Khan, Ali Khalil, Lincoln Patel, Xin J Zhou

**Affiliations:** a Transplant Services at Medical City Dallas Hospital, Dallas, TX, United States; b Interventional Radiology, Radiology Associates of North Texas, United States; c Renal Path Diagnostics at Pathologist Biomedical Laboratories, Dallas, TX, United States

**Keywords:** Kidney transplant, Renal vein thrombosis, Thrombectomy

## Abstract

**Introduction:**

Renal vein thrombosis post kidney transplant is a rare but graft threatening event. RVT is reported in 0.3–4.2% of kidney transplants. When occurring early post transplant, prior to development of collateral venous outflow, may be catastrophic with loss of the allograft or even death. Anatomic abnormalities or technical problems during surgery are common causes. Early diagnosis and urgent treatment are necessary but often unsuccessful.

Presentation of case: We report a patient with residual function in a failing allograft who developed RVT in a living donor preemptive kidney transplant.

**Discussion:**

We review the literature regarding renal vein thrombosis following kidney transplant.

**Conclusion:**

Prompt diagnosis and immediate surgicathrombectomy after ex-planting allograft with subsequent re-implanting the allograft was successful.

## Highlights

• Post transplant renal vein thrombosis is uncommon.
• Post transplant renal vein thrombosis is usually graft threatening.
• Post renal vein thrombosis rarely successfully treated.
• Early diagnosis is essential.
• Surgical thrombectomy with irrigation(TPA) of allograft after ex-plant and re-implant saved allograft.

## 1 Introduction

Renal vein thrombosis post kidney transplant is a rare but graft threatening event. Renal vein thrombosis is reported in 0.3–4.2% of kidney transplants. When occurring early post transplant, prior to development of collateral venous outflow, may be catastrophic with loss of the allograft or even death. Anatomic abnormalities or technical problems during surgery are common causes. Early diagnosis and urgent treatment are necessary but often unsuccessful. We report a patient with residual function in a failing allograft who developed renal vein thrombosis in a living donor preemptive kidney transplant. Prompt diagnosis and immediate surgical thrombectomy after ex-planting allograft with subsequent re-implanting the allograft was successful. We review the literature regarding renal vein thrombosis following kidney transplant. This work has been reported in line with SCARE criteria [9].

## 2 Presentation of case

45-year-old Caucasian woman underwent preemptive living donor kidney transplant. She had a history of end-stage renal disease secondary to type 1 diabetes mellitus and was status post living unrelated kidney transplant from a friend in August 2007. She developed polyoma virus nephropathy and acute rejection with advanced chronic kidney disease following her first transplant. She was not yet returned to dialysis when the second transplant was performed. She received induction with Thymoglobulin 4.5 mg/kg followed by maintenance immunosuppression with Tacrolimus, Mycophenolate, and Prednisone. Discharged following her second transplant with a serum creatinine of 0.9 mg/dl. Two days following her discharge she was seen in the post-transplant clinic with a serum creatinine of 1.5 mg/dl associated with 1+ lower extremity edema. Chest x-ray showed mild pulmonary congestion. Tacrolimus trough level was 9.2 ng/ml. She was prescribed Lasix 40 mg daily. She went to the emergency room the next day complaining of low-grade fever and decreased urine output. Blood pressure 123/83, pulse 110/min temperature 37.7 °C, exam unremarkable except mild pedal edema. Serum creatinine was 2 mg/dl. Doppler ultrasound revealed renal vein thrombosis with preserved arterial flow. An emergent re-exploration of the renal transplant was performed. The graft was found to have arterial inflow, but the venous system appeared completely occluded with dense thrombus. The graft was carefully explanted and additional clot was removed from the iliac vein. The graft was immediately flushed with preservation solution and tissue plasminogen activator. Next, the dense thrombus was removed from the renal vein and its segmental branches. Once all possible clots had been removed, the graft was re-implanted and a ureteral-ureterostomy was performed.

Postoperatively the patient received unfractionated intravenous heparin with careful monitoring of her PTT. Patient was seen by hematology, hypercoagulable workup was unremarkable. She remained non oliguric but her serum creatinine peaked at 6.76 mg/dl. Multiple Doppler ultrasounds revealed patency of the transplant renal artery and renal vein and a nuclear medicine renal scan showed good perfusion with delayed function of the allograft. 7 days after the surgical thrombectomy heparin was held and a needle biopsy of the transplant kidney were done which revealed significant tubular damage but no evidence of acute rejection and less than 10% tubular atrophy interstitial fibrosis).

**Fig. 1:**
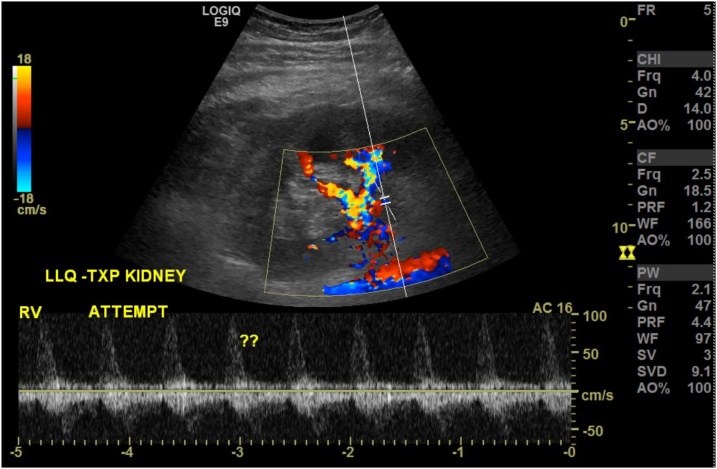
Reversal of venous flow.

**Fig. 2:**
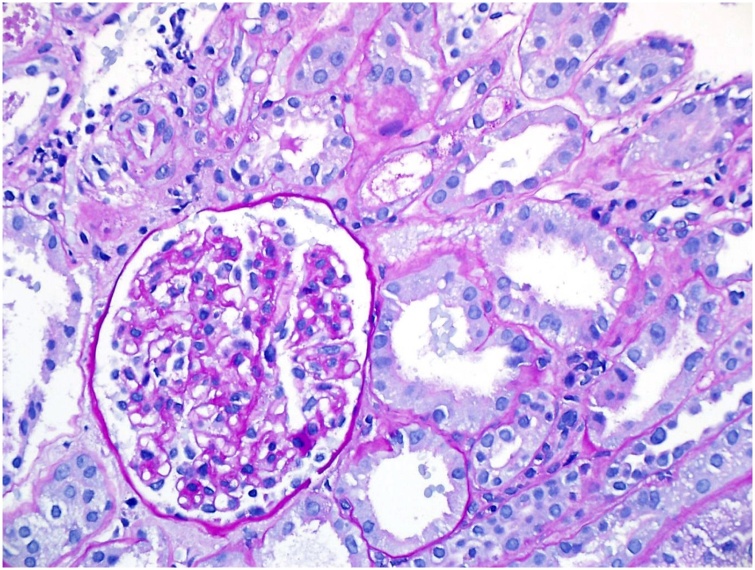
- Biopsy 1.

Triple immunosuppression was continued, eventually serum creatinine began to decrease, and at 17 weeks post renal vein thrombectomy her serum creatinine was 1.74 mg/dl (Fig. 1, Fig. 2).

## 3 Discussion

Our patient likely developed the renal vein thrombosis after discharge from the hospital with a serum creatinine of 0.9 mg/dl Three days later; her serum creatinine had increased to 1.5 mg/dl. In retrospect, the renal vein thrombosis was the cause of this increase in serum creatinine, the abnormal power Doppler documented this finding the following day. After successfully removing the thrombosis and reestablishing normal venous outflow, there was significant allograft dysfunction for several days. Serial Doppler studies demonstrating normal arterial inflow and venous outflow suggested acute tubular dysfunction. A biopsy of the allograft, ruled out acute rejection and ultimately allograft function began improving.

## 4 Conclusion

Post-transplant renal vein thrombosis is rare occurring in 0.3% to 4.2% of kidney transplant recipients. The outcome is usually poor because of the lack of collateral circulation with the venous flow originating from the renal vein [1]. This can not only lead to the loss of the graft itself but may also result in a high mortality rate due to graft rupture and embolic complications [2]. Successful revascularization with thrombolytic therapy, thrombus-aspiration, or direct surgical thrombectomy have been reported [[3], [4], [5]]. Interestingly, one case series demonstrated a significant reduction in renal vein thrombosis following kidney transplant in patients receiving low-dose Aspirin [6,7].

Power Doppler is more sensitive than color Doppler for detection of blood flow with increased confidence in the diagnosis of renal vein thrombosis [7]. Post kidney transplant renal vein thrombosis should always be in the differential diagnosis of any patient with early (less than 2 weeks) allograft dysfunction. If present, timely diagnosis is essential and gives the best chance for graft survival.

We were never able to identify an obvious precipitating cause for the renal vein thrombosis and hematologic studies were unable to document a pro-thrombotic state. No obvious anatomical precipitating factors were recognized during the initial transplant surgery. It is interesting that although the patient was initially placed on low-dose Aspirin following the transplant it was inadvertently discontinued prior to formation of the renal vein thrombosis. Our patient is currently demonstrating stable allograft function on chronic anticoagulation.

Written informed consent was obtained from the patient for publication of this case report and accompanying images. A copy of the written consent is available for review by the editor in chief of this journal on request.

## Declaration of Competing Interest

Nothing to disclose

## Sources of funding

None

## Ethical approval

Date: June 14,2019

Principal Investigatior: Mark Lerman, MD

Protocol Sponcor: N/A

Medical City Facility(ies): Medical City Dallas

Protocol Title: Post Transplant Renal Vein Thrombosis, with Successful Thrombectomy and Review of the Literature: A Case Report

NTX IRS Study #: 2019.024

## Consent

Written informed consent was obtained from the patient for publication of this case report and accompanying images. A copy of the written consent is available for review by the Editor-in-Chief of this journal on request.

## Author contribution

Mark Lerman, MD co managed patient and wrote manuscript

Matthew Mulloy, MD preformed surgical thrombectomy and Surgical description in manuscript

Christie Gooden, MD performed Kidney Transplant and Assisted with thrombectomy

Salman Khan, MD co-managed patient and reviewed manuscript

Ali Khalil, MD co managed patient and made revisions to manuscript

Lincoln Patel, MD interpreted Doppler images of renal vein thrombosis

Xin Zhou, MD interpreted biopsy of kidney transplant

## Registration of research studies

Researchregistry4743

## Guarantor

Mark Lerman,MD

Matthew Mulloy,MD

## Provenance and peer review

Not commissioned, externally peer-reviewed
